# Popular and Scientific Attitudes Regarding Pandemic Influenza

**DOI:** 10.3201/eid1409.080647

**Published:** 2008-09

**Authors:** Peter Doshi

**Affiliations:** Massachusetts Institute of Technology, Cambridge, Massachusetts, USA

**Keywords:** pandemic influenza, public opinion surveys, community mitigation measures, public health, letter

## To the Editor

Blendon et al. (*1*) described a survey of public attitudes regarding Americans’ willingness and ability to follow the advice of public health officials during a severe influenza pandemic. The authors’ results, however, can only be considered indicative if Americans’ perceptions of pandemic influenza during the next pandemic are comparable to those associated with the hypothetical event they imagined while participating in the telephone survey by Blendon et al.

By asking respondents to imagine a “severe outbreak” of “a new type of flu,” the authors likely portrayed to survey participants an image of pandemic flu as an event starkly different from ordinary flu seasons. Although such a contrast reinforces popular perceptions of pandemic flu as a catastrophic event (*2*), it is not supported by historical studies which show that, in terms of deaths, recent pandemics have been comparable to (*3*) or less deadly than (*4*) ordinary influenza seasons.

A gap thus exists between the perceptions and reality of pandemic influenza. Although the authors described pandemic flu as an “unfamiliar crisis” that “many of the respondents may not have been familiar with,” in actuality, 39% of survey respondents were >50 years of age and therefore had firsthand experience of 1 or more past pandemics. (The last 2 pandemics occurred in 1957 and 1968; a pandemic was predicted in 1976, but never materialized.) Whether those respondents were aware that they had lived through past pandemics is a question with important implications for the survey results, but unfortunately, this understanding was not queried by the authors. For example, would all of the 94% of respondents who reported a willingness to isolate themselves at home for 7–10 days if that were recommended by health authorities—in effect, “voluntarily” placing themselves in quarantine—also be willing to do so during a pandemic no more severe than ordinary influenza?

If even those who have experienced pandemics do not recall them as particularly memorable events, it calls for a rethinking of public communication strategies with respect to influenza. Perhaps a first step is to acknowledge that as the past 2 pandemics have not been public health crises, the next pandemic may likewise also not be a crisis.
