# Supplementary material for: Popular and Scientific Attitudes Regarding Pandemic Influenza
Source: Emerg Infect Dis. 2008 Sep;14(9):1501–2. doi: 10.3201/eid1409.080647 (PMC2603129; doi:10.3201/eid1409.080647)
Supplement: Related texts

# Reply: In Response

**Authors:** Robert J. Blendon, Lisa M. Koonin, John M. Benson, Martin S. Cetron, William E. Pollard, Elizabeth W. Mitchell, Kathleen J. Weldon, Melissa J. Herrmann

**Affiliations:** Harvard School of Public Health, Boston, Massachusetts, USA (R.J. Blendon, J.M. Benson, K.J. Weldon); Centers for Disease Control and Prevention, Atlanta, Georgia, USA (L.M. Koonin, M.S. Cetron, W.E. Pollard, E.W. Mitchell); International Communications Research, Media, Pennsylvania, USA (M.J. Herrmann)

We agree with Doshi (*1*) that in our study reported in “Public Response to Community Mitigation Measures for Pandemic Influenza” (*2*), we purposely asked respondents to imagine a “severe outbreak” of “a new type of flu,” and that possible scenario was vastly different from ordinary flu seasons. Although previous pandemics have varied in their severity (*3*) and their concomitant illness and mortality rates, we were particularly interested in the public’s response to community mitigation interventions (*4*) that would only be recommended if a severe 1918-like pandemic occurred (e.g., Pandemic Severity Index 4 or 5).

A great deal of cooperation from the public would be required to successfully implement community mitigation measures during a pandemic. The intensity of interventions must be matched with the severity of a pandemic to maximize the available public health benefit that may result from using these measures while minimizing untoward secondary effects. Socially disruptive measures such as dismissing children from schools, closing childcare programs, social distancing in the community and at the workplace, and cancelling large gatherings would likely reduce community transmission of pandemic disease, but would also create challenges for the public. Therefore, these interventions would only be recommended if the severity of the pandemic warranted their use. The survey was conducted to inform policy-makers who were, at the time, developing recommendations for community-based interventions. Thus, a severe pandemic was used as the scenario for this national survey to gauge the public’s response to these proposed public health measures.

## References

[1] Doshi P. Popular and scientific attitudes regarding pandemic influenza. Emerg Infect Dis. 2008;14:1501–2.1876003710.3201/eid1409.080647PMC2603129

[2] Blendon RJ, Koonin LM, Benson JM, Cetron MS, Pollard WE, Mitchell EW, Public response to community mitigation measures for pandemic influenza. Emerg Infect Dis. 2008;14:778–86.1843936110.3201/eid1405.071437PMC2600239

[3] US Department of Health and Human Services. Pandemics and pandemic threats since 1900 [cited 2008 Jun 30]. Available from http://www.pandemicflu.gov/general/historicaloverview.html

[4] Centers for Disease Control and Prevention. Interim pre-pandemic planning guidance: community strategy for pandemic influenza mitigation in the United States. 2007 Feb [cited 2008 Jun 30]. Available from http://www.pandemicflu.gov/plan/community/commitigation.html
